# HIV Care Outcomes Among Hispanics or Latinos with Diagnosed HIV Infection — United States, 2015

**DOI:** 10.15585/mmwr.mm6640a2

**Published:** 2017-10-13

**Authors:** Zanetta Gant, Andre Dailey, Xiaohong Hu, Anna Satcher Johnson

**Affiliations:** 1 Division of HIV/AIDS Prevention, National Center for HIV, Viral Hepatitis, STD, and TB Prevention, CDC.

Data from CDC’s National HIV Surveillance System (NHSS)* are used to monitor progress toward achieving national goals set forth in the Division of HIV/AIDS Prevention’s Strategic Plan (*1*) and other federal directives^†^ for human immunodeficiency virus (HIV) testing, care, and treatment outcomes and HIV-related disparities in the United States. Recent data indicate that Hispanics or Latinos^§^ are disproportionately affected by HIV infection. Hispanics or Latinos living with diagnosed HIV infection have lower levels of care and viral suppression than do non-Hispanic whites but higher levels than those reported among blacks or African Americans (*2*). The annual rate of diagnosis of HIV infection among Hispanics or Latinos is three times that of non-Hispanic whites (*3*), and a recent study found increases in incidence of HIV infection among Hispanic or Latino men who have sex with men (*4*). Among persons with HIV infection diagnosed through 2013 who were alive at year-end 2014, 70.2% of Hispanics or Latinos received any HIV medical care compared with 76.1% of non-Hispanic whites (*2*). CDC used NHSS data to describe HIV care outcomes among Hispanics or Latinos. Among male Hispanics or Latinos with HIV infection diagnosed in 2015, fewer males with infection attributed to heterosexual contact (34.6%) had their infection diagnosed at an early stage (stage 1 = 12.0%, stage 2 = 22.6%) than males with infection attributed to male-to-male sexual contact (60.9%: stage 1 = 25.2%, stage 2 = 35.7%). The percentage of Hispanics or Latinos linked to care after diagnosis of HIV infection increased with increasing age; females aged 45–54 years with infection attributed to injection drug use (IDU) accounted for the lowest percentage (61.4%) of persons linked to care. Among Hispanics or Latinos living with HIV infection, care and viral suppression were lower among selected age groups of Hispanic or Latino males with HIV infection attributed to IDU than among males with infection attributed to male-to-male sexual contact and male-to-male sexual contact and IDU. Intensified efforts to develop and implement effective interventions and public health strategies that increase engagement in care and viral suppression among Hispanics or Latinos (*3*,*5*), particularly those who inject drugs, are needed to achieve national HIV prevention goals.

All states, the District of Columbia, and U.S. territories report cases of HIV infection and associated demographic and clinical information to NHSS. CDC analyzed data for persons aged ≥13 years reported through December 2016 from 38 jurisdictions^¶^ with complete laboratory reporting.** These jurisdictions accounted for 75.2% of Hispanics or Latinos aged ≥13 years living with diagnosed HIV infection at year-end 2014 in the United States. Stage of disease at diagnosis and linkage to care were assessed among Hispanics or Latinos living in any of the 38 jurisdictions at the time of diagnosis of HIV infection in 2015. For persons who received a diagnosis of HIV infection during 2015, linkage to HIV care within 1 month of diagnosis was measured by documentation of one or more CD4 (cell count or percentage) or viral load tests performed ≤1 month after diagnosis of HIV infection, including tests performed on the date of diagnosis. Receipt of any HIV care, defined as having one or more CD4 or viral load tests during 2014, retention in care, defined as having two or more CD4 or viral load tests ≥3 months apart, and viral suppression, defined as a viral load of <200 HIV RNA copies/mL at most recent test (*6*) were assessed among Hispanics or Latinos with HIV infection diagnosed by December 31, 2013, and who were alive and resided (based on the most recent known address) in any of the 38 jurisdictions as of December 31, 2014 (i.e., persons living with diagnosed HIV infection). Data were statistically adjusted by using multiple imputation techniques to account for missing HIV transmission categories (*3*).

In the 38 jurisdictions, 6,707 Hispanics or Latinos received a diagnosis of HIV infection in 2015 (Table 1). Among these persons, 24.5% had infection classified as stage 1 at diagnosis, 33.6% as stage 2, and 23.1% as stage 3 (acquired immunodeficiency syndrome [AIDS]); for 18.8% the stage was unknown (*6*). Among both males and females, the highest percentage of infections were diagnosed at an earlier stage (stage 1 [24.5%] or stage 2 [33.6%]). By age group, the highest percentage of Hispanics or Latinos whose infection was diagnosed at stage 1 or stage 2 was reported in persons aged 13–24 years (stage 1, 30.8%; stage 2, 41.1%), followed by persons aged 25–34 years (stage 1, 24.3%; stage 2, 36.8%). In general, the percentages of early diagnosis decreased as age increased; among persons aged ≥55 years, 40.1% of infections were diagnosed at stage 3 (Table 1). By transmission category, the highest percentages of Hispanics or Latinos with infection diagnosed at an earlier stage of HIV disease were among females with infection attributed to heterosexual contact (stage 1, 27.2%; stage 2, 29.2%). Males with infection attributed to heterosexual contact accounted for the lowest percentages of early diagnoses (stage 1, 12.0%; stage 2, 22.6%).

**TABLE 1:** Number of diagnoses of HIV infection among Hispanics or Latinos aged ≥13 years, by stage of disease* — National HIV Surveillance System, 38 jurisdictions,^†^ United States, 2015

Characteristic	Total	Stage 1 (CD4 ≥500 cells/*μ*L or ≥26%)	Stage 2 (CD4 200–499 cells/*μ*L or 14%–25%)	Stage 3 (AIDS) (CD4 <200 cells/*μ*L or <14%)	Stage unknown (no CD4 information)
		No. (%)	No. (%)	No. (%)	No. (%)
**Sex**					
Male	**5,925**	1,437 (24.3)	2,027 (34.2)	1,351 (22.8)	1,110 (18.7)6224
Female	**782**	208 (26.6)	225 (28.8)	196 (25.1)	153 (19.6)
**Age group at diagnosis (yrs)**					
13–24	**1,509**	465 (30.8)	620 (41.1)	133 (8.8)	291 (19.3)
25–34	**2,397**	583 (24.3)	883 (36.8)	453 (18.9)	478 (19.9)
35–44	**1,482**	320 (21.6)	419 (28.3)	484 (32.7)	259 (17.5)
45–54	**918**	199 (21.7)	235 (25.6)	316 (34.4)	168 (18.3)
≥55	**401**	78 (19.5)	95 (23.7)	161 (40.1)	67 (16.7)
**Transmission category^§^**					
Male-to-male sexual contact	**5,124**	1,289 (25.2)	1,831 (35.7)	1,033 (20.2)	971 (18.9)
**Injection drug use**					
Male	**237**	49 (20.8)	57 (24.1)	78 (33.0)	53 (22.1)
Female	**96**	21 (22.3)	25 (25.9)	25 (26.5)	24 (25.3)
Male-to-male sexual contact and injection drug use	**212**	55 (26.1)	61 (28.7)	54 (25.4)	42 (19.8)
**Heterosexual contact^¶^**					
Male	**345**	42 (12.0)	78 (22.6)	181 (52.5)	44 (12.8)
Female	**684**	186 (27.2)	200 (29.2)	171 (24.9)	128 (18.7)
Total**	**6,707**	**1,645 (24.5)**	**2,252 (33.6)**	**1,547 (23.1)**	**1,263 (18.8)**

**Abbreviations:** AIDS = acquired immunodeficiency syndrome; HIV = human immunodeficiency virus.

* Stage of disease at diagnosis of HIV infection based on first CD4 test performed or documentation of an AIDS-defining condition ≤3 months after a diagnosis of HIV infection. Selik RM, Mokotoff ED, Branson B, Owen SM, Whitmore S, Hall HI. Revised surveillance case definition for HIV infection—United States, 2014. MMWR Recomm Rep 2014;63(No. RR-03).

^†^ The 38 jurisdictions were Alabama, Alaska, California, Colorado, Connecticut, Delaware, District of Columbia, Georgia, Hawaii, Illinois, Indiana, Iowa, Louisiana, Maine, Maryland, Massachusetts, Michigan, Minnesota, Mississippi, Missouri, Montana, Nebraska, New Hampshire, New Mexico, New York, North Dakota, Oregon, Rhode Island, South Carolina, South Dakota, Tennessee, Texas, Utah, Virginia, Washington, West Virginia, Wisconsin, and Wyoming.

^§^ Data statistically adjusted using multiple imputation techniques to account for missing transmission categories.

^¶^ Heterosexual contact with a person known to have, or to be at high risk for, HIV infection.

** Includes persons with diagnosed infection attributed to hemophilia, blood transfusion, perinatal exposure, and risk factors not reported or not identified.

Overall, 5,059 (75.4%) of the 6,707 Hispanics or Latinos with HIV infection diagnosed during 2015 were linked to care within 1 month of diagnosis; the percentage of persons linked to care increased with increasing age (Table 2). By transmission category and age group, males aged ≥55 years with infection attributed to heterosexual contact accounted for the highest percentage of persons linked to care (88.0%), whereas females aged 45–54 years with infection attributed to IDU accounted for the lowest percentage (61.4%).

**TABLE 2:** Number of persons linked to HIV medical care within 1 month after diagnosis of HIV infection among Hispanics or Latinos aged ≥13 years, by age group and selected characteristics — National HIV Surveillance System, 38 jurisdictions,* United States, 2015

Characteristic	13–24 yrs		25–34 yrs		35–44 yrs		45–54 yrs		≥55 yrs		Total	
	No. HIV diagnoses	No. linked^†^ (%)	No. HIV diagnoses	No. linked^†^ (%)	No. HIV diagnoses	No. linked^†^ (%)	No. HIV diagnoses	No. linked^†^ (%)	No. HIV diagnoses	No. linked^†^ (%)	No. HIV diagnoses	No. linked^†^ (%)
**Sex**												
Male	1,375	1,000 (72.7)	2,197	1,639 (74.6)	1,289	995 (77.2)	760	594 (78.2)	304	241 (79.3)	**5,925**	**4,469 (75.4)**
Female	134	100 (74.6)	200	142 (71.0)	193	147 (76.2)	158	121 (76.6)	97	80 (82.5)	**782**	**590 (75.4)**
**Transmission category^§^**												
Male-to-male sexual contact	1,279	930 (72.7)	1,971	1,465 (74.4)	1,068	826 (77.4)	615	476 (77.4)	192	148 (77.3)	**5,124**	**3,845 (75.0)**
**Injection drug use**												
Male	23	16 (69.2)	59	43 (72.1)	62	42 (67.8)	52	39 (75.3)	42	31 (73.5)	**237**	**170 (71.6)**
Female	16	11 (69.9)	22	14 (63.8)	23	17(76.1)	23	14 (61.4)	12	9 (81.2)	**96**	**67 (69.3)**
Male-to-male sexual contact and injection drug use	51	37 (73.2)	85	61 (70.8)	47	35(74.7)	23	19 (82.7)	6	6 (100)	**212**	**157 (74.2)**
**Heterosexual contact^¶^**												
Male	22	16 (75.2)	80	69 (86.1)	111	90 (81.6)	71	60 (85.3)	62	54 (88.0)	**345**	**290 (84.1)**
Female	117	88 (75.2)	178	128 (72.0)	170	130 (76.2)	135	107 (79.2)	85	70 (82.6)	**684**	**522 (76.3)**
Other**												
Male	1	1 (100)	2	2 (100)	2	2 (100)	1	1 (100)	2	2 (100)	**7**	**7 (100)**
Female	1	1 (100)	0	0 (0.0)	0	0 (0.0)	0	0 (0.0)	0	0 (0.0)	**1**	**1 (100)**
**Total**	**1,509**	**1,100** (**72.9**)	**2,397**	**1,781 (74.3)**	**1,482**	**1,142 (77.1)**	**918**	**715 (77.9)**	**401**	**321 (80)**	**6,707**	**5,059 (75.4)**

**Abbreviation:** HIV = human immunodeficiency virus.

* The 38 jurisdictions were Alabama, Alaska, California, Colorado, Connecticut, Delaware, District of Columbia, Georgia, Hawaii, Illinois, Indiana, Iowa, Louisiana, Maine, Maryland, Massachusetts, Michigan, Minnesota, Mississippi, Missouri, Montana, Nebraska, New Hampshire, New Mexico, New York, North Dakota, Oregon, Rhode Island, South Carolina, South Dakota, Tennessee, Texas, Utah, Virginia, Washington, West Virginia, Wisconsin, and Wyoming.

^†^ One or more CD4 or viral load tests performed within 1 month after HIV diagnosis during 2015.

^§^ Data statistically adjusted using multiple imputation techniques to account for missing transmission categories.

^¶^ Heterosexual contact with a person known to have, or to be at high risk for, HIV infection.

** Includes persons with diagnosed infection attributed to hemophilia, blood transfusion, perinatal exposure, and risk factors not reported or not identified.

Among 141,929 Hispanics or Latinos aged ≥13 years living with diagnosed HIV infection in 38 jurisdictions in 2015, 70.2% received care, and 58.3% were retained in care (Table 3), with males having lower receipt of care (69.0%) and retention in care (57.1%) than females (74.6% and 63.0%, respectively). By transmission category and age group, males aged 25–34 years with infection attributed to IDU accounted for the lowest percentages of persons who received (55.7%) and were retained (44.8%) in care. At the most recent test, 58.2% of Hispanics or Latinos had suppressed viral load (Table 3); a higher percentage of females had suppressed viral load (59.7%) than did males (57.8%). Among all age groups, the lowest level of viral load suppression was among persons aged 13–24 years (54.6%); viral load suppression increased with increasing age. Males aged 25–34 years and 35–44 years with infection attributed to IDU had the lowest levels of viral suppression (38.9% and 43.1%, respectively).

**TABLE 3:** Receipt of HIV medical care and viral suppression among Hispanics or Latinos aged ≥13 years with HIV infection diagnosed by December 31, 2013,* who were alive on December 31, 2014, by age group and selected characteristics — National HIV Surveillance System, 38 jurisdictions,^†^ United States, 2015

Characteristic	Total no.	Receipt of HIV care in 2014		Viral suppression**
		Any care^§^	Retained in care^¶^	
		No. (%)	No. (%)	No. (%)
**Age ≥13 yrs^††^**				
**Sex**				
Male	113,284	78,214 (69.0)	64,661 (57.1)	65,532 (57.8)
Female	28,645	21,375 (74.6)	18,048 (63.0)	17,108 (59.7)
**Transmission category^§§^**				
Male-to-male sexual contact	79,146	56,407 (71.3)	46,256 (58.4)	48,027 (60.7)
**Injection drug use**				
Male	15,733	9,026 (57.4)	7,770 (49.4)	7,318 (46.5)
Female	7,244	5,269 (72.7)	4,510 (62.3)	4,064 (56.1)
Male-to-male sexual contact and injection drug use	8,086	5,982 (74.0)	4,981 (61.6)	4,636 (57.3)
**Heterosexual contact^¶¶^**				
Male	9,143	5,967 (65.3)	4,983 (54.5)	4,974 (54.4)
Female	20,303	15,259 (75.2)	12,831 (63.2)	12,495 (61.5)
Other***	2,274	1,678 (77.1)	1,378 (60.6)	1,126 (49.5)
**Total**	**141,929**	**99,589 (70.2)**	**82,709 (58.3)**	**82,640 (58.2)**
**Age 13–24 yrs^††^**				
**Sex**				
Male	4,493	3,466 (77.1)	2,689 (59.8)	2,530 (56.3)
Female	1,298	1,000 (77)	817 (62.9)	631 (48.6)
**Transmission category^§§^**				
Male-to-male sexual contact	3,558	2,767 (77.8)	2,116 (59.5)	2,081 (58.5)
**Injection drug use**				
Male	51	40 (77.1)	31 (61.1)	25 (48.8)
Female	64	45 (70.4)	37 (58.4)	30 (46.5)
Male-to-male sexual contact and injection drug use	162	123 (75.8)	96 (59.1)	71 (43.5)
**Heterosexual contact^¶¶^**				
Male	78	55 (70.9)	47 (60.4)	42 (53.8)
Female	514	388 (75.6)	310 (60.4)	257 (50.0)
Other***	1,365	1,048 (76.8)	869 (63.3)	656 (48.1)
**Subtotal**	**5,791**	**4,466 (77.1)**	**3,506 (60.5)**	**3,161 (54.6)**
**Age 25–34 yrs^††^**				
**Sex**				
Male	19,983	14,229 (71.2)	11,138 (55.7)	11,191 (56.0)
Female	3,855	2,752 (71.4)	2,172 (56.3)	2,007 (52.1)
**Transmission category^§§^**				
Male-to-male sexual contact	16,715	12,054 (72.1)	9,416 (56.3)	9,637 (57.7)
**Injection drug use**				
Male	713	398 (55.7)	320 (44.8)	278 (38.9)
Female	538	388 (72.0)	295 (54.8)	251 (46.6)
Male-to-male sexual contact and injection drug use	1,201	917 (76.3)	735 (61.2)	626 (52.1)
**Heterosexual contact^¶¶^**				
Male	1,052	660 (62.7)	512 (48.7)	517 (49.1)
Female	3,077	2,179 (70.8)	1,723 (56.0)	1,631 (53.0)
Other***	542	386 (77.1)	309 (57.0)	259 (47.8)
**Subtotal**	**23,838**	**16,981 (71.2)**	**13,310 (55.8)**	**13,198 (55.4)**
**Age 35–44 yrs^††^**				
**Sex**				
Male	29,744	20,299 (68)	16,476 (55.4)	16,921 (56.9)
Female	7,253	5,343 (74)	4,341 (59.9)	4,131 (57.0)
**Transmission category^§§^**				
Male-to-male sexual contact	22,581	15,780 (70)	12,846 (56.9)	13,419 (59.4)
**Injection drug use**				
Male	2,468	1,399 (57)	1,131 (45.8)	1,063 (43.1)
Female	1,521	1,108 (73)	883 (58.1)	787 (51.7)
Male-to-male sexual contact and injection drug use	2,145	1,551 (72)	1,236 (57.7)	1,185 (55.2)
**Heterosexual contact^¶¶^**				
Male	2,482	1,518 (61)	1,225 (49.4)	1,212 (48.8)
Female	5,714	4,222 (74)	3,446 (60.3)	3,333 (58.3)
Other***	86	65 (77.8)	50 (58.1)	53 (61.6)
**Subtotal**	**36,997**	**25,642 (69.3)**	**20,817 (56.3)**	**21,052 (56.9)**
**Age 45–54 yrs^††^**				
**Sex**				
Male	37,491	26,015 (69.4)	21,894 (58.4)	22,281 (59.4)
Female	9,436	7,192 (76.2)	6,201 (65.7)	5,882 (62.3)
**Transmission category^§§^**				
Male-to-male sexual contact	24,927	17,898 (71.8)	15,008 (60.2)	15,703 (63.0)
**Injection drug use**				
Male	6,253	3,696 (59.1)	3,165 (50.6)	2,959 (47.3)
Female	3,008	2,237 (74.4)	1,961 (65.2)	1,740 (57.9)
Male-to-male sexual contact and injection drug use	3,094	2,294 (74.2)	1,947 (62.9)	1,834 (59.3)
**Heterosexual contact^¶¶^**				
Male	3,146	2,080 (66.1)	1,737 (55.2)	1,741 (55.4)
Female	6,384	4,924 (77.1)	4,213 (66.0)	4,115 (64.5)
Other***	115	78 (70.5)	63 (54.8)	68 (59.1)
**Subtotal**	**46,927**	**33,207 (70.8)**	**28,095 (59.9)**	**28,163 (60.0)**
**Age ≥55 yrs^††^**				
**Sex**				
Male	21,573	14,205 (65.8)	12,464 (57.8)	12,609 (58.4)
Female	6,803	5,088 (74.8)	4,517 (66.4)	4,457 (65.5)
**Transmission category^§§^**				
Male-to-male sexual contact	11,364	7,908 (69.6)	6,870 (60.5)	7,186 (63.2)
**Injection drug use**				
Male	6,248	3,495 (55.9)	3,123 (50.0)	2,993 (47.9)
Female	2,113	1,492 (70.6)	1,334 (63.1)	1,256 (59.4)
Male-to-male sexual contact and injection drug use	1,485	1,098 (73.9)	967 (65.1)	921 (62.0)
**Heterosexual contact^¶¶^**				
Male	2,385	1,653 (69.3)	1,461 (61.3)	1,462 (61.3)
Female	4,615	3,546 (76.8)	3,139 (68.0)	3,159 (68.4)
Other***	166	102 (68.0)	88 (53.0)	90 (54.2)
**Subtotal**	**28,376**	**19,293 (68.0)**	**16,981 (59.8)**	**17,066 (60.1)**

**Abbreviation:** HIV = human immunodeficiency virus.

* Data are based on address of residence as of December 31, 2014 (i.e., most recent known address). Hispanics or Latinos might be of any race.

^†^ The 38 jurisdictions were Alabama, Alaska, California, Colorado, Connecticut, Delaware, the District of Columbia, Georgia, Hawaii, Illinois, Indiana, Iowa, Louisiana, Maine, Maryland, Massachusetts, Michigan, Minnesota, Mississippi, Missouri, Montana, Nebraska, New Hampshire, New Mexico, New York, North Dakota, Oregon, Rhode Island, South Carolina, South Dakota, Tennessee, Texas, Utah, Virginia, Washington, West Virginia, Wisconsin, and Wyoming.

^§^ Defined as having at least one CD4 or VL test performed during 2014, among persons diagnosed through December 31, 2013, and alive on December 31, 2014.

^¶^ Defined as having two or more CD4 or VL tests performed ≥3 months apart during 2014, among persons diagnosed through December 31, 2013, and alive on December 31, 2014.

** Defined as having a VL result of ≤200 copies/mL at the most recent VL test during 2014. The cut-off value of ≤200 copies/mL was based on the U.S. Department of Health and Human Services recommended definition of virologic failure. https://aidsinfo.nih.gov/guidelines/html/1/adult-and-adolescent-arv-guidelines/15/virologic-failure.

^††^ Age at year-end 2014.

^§§^ Data statistically adjusted using multiple imputation techniques to account for missing transmission categories.

^¶¶^ Heterosexual contact with a person known to have or to be at high risk for HIV infection.

*** Includes persons with diagnosed infection attributed to hemophilia, blood transfusion, perinatal exposure, and risk factor not reported or not identified.

## Discussion

In 2015, among Hispanics or Latinos aged ≥13 years with diagnosed HIV infection in 38 jurisdictions with complete laboratory reporting, 58.1% of infections were diagnosed at an earlier stage (stage 1 or 2) and another 18.8% at an unknown stage; overall, 75.4% were linked to care within 1 month of diagnosis. Among all Hispanics or Latinos aged ≥13 years living with diagnosed HIV infection at year-end 2014 in these jurisdictions, 58.3% were retained in care, and 58.2% had suppressed viral load. By comparison, the national goals are 85% linkage to care, 90% retention in care, and 80% viral load suppression (*1*), and the percentages among non-Hispanic whites were 79.9%, 58.5%, and 65.0%, respectively (*3*). Improving health outcomes for Hispanics or Latinos living with HIV infection is necessary to reduce HIV transmission in the United States. Prompt linkage to care after diagnosis allows early initiation of HIV treatment, which is associated with reduced morbidity, mortality, and transmission of HIV infection (*7*).

Consistent with findings from a previous report on the continuum of HIV care among Hispanics or Latinos with diagnosed HIV infection based on data from 19 jurisdictions, linkage to care was similar for both males and females, retention in care followed a similar pattern across age groups, and males had lower levels of viral suppression than did females (*8*). The lowest levels of care and viral suppression among Hispanics or Latinos with HIV infection in these 38 jurisdictions were among males with infection attributed to IDU, and the highest levels of care and viral suppression were among heterosexual females. Hispanics or Latinos in the four age groups ≥25 years had similar percentages of retention in care and viral suppression. Those aged 13–24 years had the highest retention in care among all age groups (60.5%) and the lowest viral suppression (54.6%); the reasons for this are not known. Hispanics or Latinos with HIV infection might not seek, receive, or adhere to HIV care or treatment regimens for various reasons, including lack of health insurance, language barriers, and migration patterns (*9*). HIV programs that focus on care and treatment for Hispanics or Latinos might consider strengthening efforts to link to and retain in care persons with HIV infection and to promote adherence to medication to achieve optimal health outcomes.

The findings in this report are subject to at least two limitations. First, analyses were limited to 38 jurisdictions with complete laboratory reporting of all levels of CD4 and viral load test results; these 38 jurisdictions might not be representative of all Hispanics or Latinos living with diagnosed HIV infection in the United States. Second, comparisons of numbers and percentages by sex, transmission category, and age group should be interpreted with caution because groups vary in size and some have small, unstable numbers. Reported numbers smaller than 12 and their accompanying percentages also should be interpreted with caution.

Increasing the proportion of Hispanics or Latinos living with HIV infection who receive optimal HIV care will help achieve the national goal of reducing racial/ethnic disparities in HIV care outcomes. Through partnerships with federal, state, and local health agencies, CDC is pursuing a high-impact prevention approach to maximize the effectiveness of current HIV prevention and care methods (*10*). CDC supports projects focused on Hispanics or Latinos to optimize outcomes along the HIV care continuum, such as HIV testing (the first essential step for entry into the continuum of care) and projects that support linkage to, retention in, and return to care for all persons with HIV infection.^††^ Among Hispanics or Latinos, targeting strategies to groups that bear a disproportionate burden of HIV disease (e.g., persons who inject drugs) could lead to reductions in HIV infections and health inequities and help achieve the national goal of 80% of all persons living with HIV infection having a suppressed viral load.

**Summary**

**What is already known about this topic?**

Hispanics or Latinos living with diagnosed human immunodeficiency virus (HIV) infection have lower levels of care and viral suppression than do non-Hispanic whites but higher levels than those reported among blacks or African Americans. National goals include 85% linkage to care, 90% retention in care, and 80% viral load suppression by 2020.

**What is added by this report?**

In 2015, 58.1% of HIV infections among Hispanics or Latinos aged ≥13 years with diagnosed HIV infection in 38 jurisdictions with complete laboratory reporting were diagnosed at an earlier stage (stage 1 or 2) and another 18.8% at an unknown stage; 75.4% were linked to care within 1 month of diagnosis. Among Hispanics or Latinos living with diagnosed HIV infection at year-end 2014, 70.2% received care, 58.3% were retained in care, and 58.2% were virally suppressed. The lowest levels of care and viral suppression were among males with infection attributed to injection drug use, and the highest levels of care and viral suppression were among heterosexual females. Hispanics or Latinos in the four age groups ≥25 years had similar percentages of retention and viral suppression. Those aged 13–24 years had the highest retention in care among all age groups (60.5%), but had the lowest overall viral suppression (54.6%).

**What are the implications for public health practice?**

Increasing the proportion of Hispanics or Latinos living with HIV infection who are receiving care and treatment will help to achieve the national goals to reduce new infections, improve health outcomes, and decrease health disparities. Among Hispanics or Latinos, targeted strategies for different groups, such as persons who inject drugs, might be needed to achieve improvements in linkage, care, and viral suppression.

## Footnotes

* NHSS is the primary source for monitoring HIV trends in the United States. The system collects, analyzes, and disseminates information about new and existing cases of HIV infection.

† The national goals to be achieved by 2020 are 1) 85% of all persons with newly diagnosed HIV infection to be linked to care, 2) 90% of persons living with diagnosed HIV infection to be retained in care, and 3) 80% of persons living with diagnosed HIV infection to have a suppressed viral load.

§ Hispanics or Latinos might be of any race.

¶ The 38 jurisdictions were Alabama, Alaska, California, Colorado, Connecticut, Delaware, District of Columbia, Georgia, Hawaii, Illinois, Indiana, Iowa, Louisiana, Maine, Maryland, Massachusetts, Michigan, Minnesota, Mississippi, Missouri, Montana, Nebraska, New Hampshire, New Mexico, New York, North Dakota, Oregon, Rhode Island, South Carolina, South Dakota, Tennessee, Texas, Utah, Virginia, Washington, West Virginia, Wisconsin, and Wyoming.

** The criteria for complete reporting were as follows: 1) the jurisdiction’s laws or regulations required reporting of all CD4 and viral load test results to the state or local health department, 2) ≥95% of all laboratory test results were reported by laboratories that conduct HIV-related testing for each jurisdiction, and 3) the jurisdiction reported to CDC ≥95% of CD4 and viral load results received since at least January 2014.

†† https://www.cdc.gov/hiv/research/demonstration/capus/index.html.
